# Non-Homologous End-Joining Pathway Associated with Occurrence of Myocardial Infarction: Gene Set Analysis of Genome-Wide Association Study Data

**DOI:** 10.1371/journal.pone.0056262

**Published:** 2013-02-15

**Authors:** Jeffrey J. W. Verschuren, Stella Trompet, Joris Deelen, David J. Stott, Naveed Sattar, Brendan M. Buckley, Ian Ford, Bastiaan T. Heijmans, Henk-Jan Guchelaar, Jeanine J. Houwing-Duistermaat, P. Eline Slagboom, J. Wouter Jukema

**Affiliations:** 1 Department of Cardiology, Leiden University Medical Center, Leiden, The Netherlands; 2 Department of Geriatrics and Gerontology, Leiden University Medical Center, Leiden, The Netherlands; 3 Molecular Epidemiology, Leiden University Medical Center, Leiden, The Netherlands; 4 Academic Section of Geriatric Medicine, University of Glasgow, Glasgow, United Kingdom; 5 Institute of Cardiovascular and Medical Sciences, University of Glasgow, Glasgow, United Kingdom; 6 Department of Pharmacology and Therapeutics, Cork University Hospital, Cork, Ireland; 7 Robertson Centre for Biostatistics, University of Glasgow, Glasgow, United Kingdom; 8 Department of Clinical Pharmacy & Toxicology, Leiden University Medical Center, Leiden, The Netherlands; 9 Department of Medical Statistics and Bioinformatics, Leiden University Medical Center, Leiden, The Netherlands; 10 Netherlands Consortium for Healthy Ageing, Leiden University Medical Center, Leiden, The Netherlands; 11 Durrer Center for Cardiogenetic Research, Amsterdam, The Netherlands; 12 The Interuniversity Cardiology Institute (ICIN), Utrecht, The Netherlands; Saint Louis University, United States of America

## Abstract

**Purpose:**

DNA repair deficiencies have been postulated to play a role in the development and progression of cardiovascular disease (CVD). The hypothesis is that DNA damage accumulating with age may induce cell death, which promotes formation of unstable plaques. Defects in DNA repair mechanisms may therefore increase the risk of CVD events. We examined whether the joints effect of common genetic variants in 5 DNA repair pathways may influence the risk of CVD events.

**Methods:**

The PLINK set-based test was used to examine the association to myocardial infarction (MI) of the DNA repair pathway in GWAS data of 866 subjects of the GENetic DEterminants of Restenosis (GENDER) study and 5,244 subjects of the PROspective Study of Pravastatin in the Elderly at Risk (PROSPER) study. We included the main DNA repair pathways (base excision repair, nucleotide excision repair, mismatch repair, homologous recombination and non-homologous end-joining (NHEJ)) in the analysis.

**Results:**

The NHEJ pathway was associated with the occurrence of MI in both GENDER (P = 0.0083) and PROSPER (P = 0.014). This association was mainly driven by genetic variation in the *MRE11A* gene (P_GENDER_ = 0.0001 and P_PROSPER_ = 0.002). The homologous recombination pathway was associated with MI in GENDER only (P = 0.011), for the other pathways no associations were observed.

**Conclusion:**

This is the first study analyzing the joint effect of common genetic variation in DNA repair pathways and the risk of CVD events, demonstrating an association between the NHEJ pathway and MI in 2 different cohorts.

## Introduction

Cardiovascular disease (CVD) is caused by interplay of environmental factors and multiple predisposing genes. DNA damage, caused by for instance oxidative stress and cigarette smoking, has been recognized as a significant contributor to the pathogenesis of CVD. [1], [2] Mechanistically, in cells where the DNA damage is beyond repair apoptosis is induced. [3] The effect of this damage induced cell death is dependent on the cell type. Death of endothelial cells is implicated in plaque erosion and subsequent vessel thrombosis. [4] Vascular smooth muscle cell (VSMC) death has been associated with thinning of the fibrous cap and increasing the risk of plaque rupture. [5], [6] To further complicate matters, apoptosis is not the only response of cells to DNA damage, also cellular senescence has been described. [7], [8] Cellular senescence is a state in which cells remain in cell cycle arrest and in which they have lost their optimal function. With respect to the pathogenesis of atherosclerosis, senescence of for instance vascular endothelial cells can result in a provasoconstrictor and a proinflammatory phenotype. [7] So besides cell death, DNA damage could also increases the risk of CVD by inducing cell senescence.

Adequate DNA repair is crucial for survival of an organism, as the DNA is continuously exposed to various types of external factors, like mutagenic chemicals and radiation, and endogenously generated triggers like reactive oxygen species (ROS) and DNA replication errors, all capable of inducing DNA damage. Human cells possess several innate DNA repair processes to protect against the harmful consequences of DNA damage. [9] Single-strand DNA damage can be repaired by excision repair and mismatch repair pathways that use the undamaged strand as template during the repair process. For the repair of double-strand breaks other repair mechanisms like non-homologous end-joining (NHEJ) or homologous recombination are required. [10].

Evidence of the relation between genomic integrity and cardiovascular disease in the ageing population has been growing over the last years. Up to now, this evidence consists in various forms, ranging from cellular biology studies in vascular endothelial cells [11], [12], vascular smooth muscle cells [13], [14] and macropahges [15], histological examination of human atherosclerotic plaques [14], [16] and animal studies in telomerase deficient mice [17] and DNA repair defective mice. [8], [18], [19] In contrast, only limited studies have focused on single nucleotide polymorphisms (SNPs) in genes related to DNA repair processes and CVD events, although some associations have been reported. The only SNP with some consistent results is the Arg399Gln (rs25487) SNP in the *XRCC1* base excision repair (BER) gene which was reported to be associated with stroke [20] and coronary atherosclerosis [21]. Other genes from the excision repair pathway such as *OGG1*, *XRCC3*, *ERCC2* (*XPD*) and *ERCC5*, were found to moderately associated as a combined score to the risk of large artery atherosclerotic stroke in the smoking subset of a Chinese population [22]. The authors suggested that the individual vulnerability to smoking-induced oxidative stress was influenced by carriers of these SNPS.

In genome-wide association studies (GWAS) investigating the genetic background of CVD, no association was found with SNPs in genes related to DNA repair processes. [23], [24] However, considering the multifactorial nature of the condition, it is possible that by a joint effect, genetic variants with small individual effect sizes, could contribute to disease risk and are undetected in a GWAS. [25], [26] The goal of the current study was to examine whether common genetic variants in DNA repair genes are related to the risk of CVD events by using a gene set analysis of the 5 main DNA repair pathways in two large representative CVD populations.

## Methods

### GENDER Study Population

The design of the GENetic DEterminants of Restenosis (GENDER) study has been described previously. [27] In brief, GENDER included 3,104 consecutive unrelated symptomatic patients treated successfully by PCI for angina. The study protocol conforms to the Declaration of Helsinki and was approved by the ethics committees of each participating institution. Written informed consent was obtained from each participant before the PCI procedure. Experienced operators, using a radial or femoral approach, performed standard angioplasty and stent placement. During the study, no drug-eluting stents were used. Blood samples were collected at the index procedure for DNA isolation. During a follow-up period of 9 months, the endpoint clinical restenosis, defined as renewed symptoms requiring target vessel revascularization (TVR) either by repeated PCI or CABG, by death from cardiac causes or myocardial infarction (MI) not attributable to another coronary event than the target vessel, was recorded. Furthermore, of each patient the occurrence of MI or stroke prior to inclusion into the study, as well as during the follow up period, was recorded. For this study the combination of prevalent and incident MI or stroke was analyzed.

### PROSPER Study Population

The design and population of the PROSPective study for the Elderly at Risk (PROSPER) has been described previously. [28] PROSPER is a prospective multicenter randomized placebo-controlled trial to assess whether treatment with pravastatin diminishes the risk of major vascular events in elderly individuals. Between December 1997 and May 1999, subjects were screened and enrolled in Scotland (Glasgow), Ireland (Cork), and The Netherlands (Leiden). Men and women aged 70–82 years were recruited if they had pre-existing vascular disease or increased risk of such disease because of smoking, hypertension, or diabetes. A total number of 5,804 subjects were randomly assigned to pravastatin or placebo. In this study several cardiovascular endpoints were evaluated during a mean follow-up of 3.2 years; the primary endpoint consisted of a composite of fatal/non-fatal MI or fatal/non-fatal stroke. Secondary and tertiary endpoints included stroke and MI separately, all-cause mortality and death due to a vascular cause. [29] The institutional ethics review boards of all centers approved the protocol, and all participants gave written informed consent. The protocol was consistent with the Declaration of Helsinki. For the current study we combined the incident events,myocardial infarction and stroke, that occurred during the follow-up period with the prevalent events that occurred before inclusion into PROSPER, to obtain a lifetime risk for these events.

### Genotyping

In GENDER, a GWAS was performed in 325 cases of restenosis and 630 controls matched by gender, age, and some confounding clinical variables for restenosis in the GENDER study such as total occlusion, diabetes, current smoking and residual stenosis. [30] Genotyping was performed using the Illumina Human 610-Quad Beadchips following manufacturer’s instructions. After stringent quality control, bad performing samples (call rate <99%) and assays (call rate <95%, minor allele frequency <1% and deviation from Hardy-Weinberg equilibrium) were excluded from further analysis. The final dataset consisted of 866 individuals (295 cases, 571 controls) and 556,099 SNPs.

In PROSPER, a GWAS was performed using Illumina Human 660-Quad Beadchips following manufacturer’s instructions. After stringent quality control, bad performing samples and assays were excluded from further analysis. Genotypic data was available in 5,244 subjects and a total of 557,192 SNPs [31].

Both datasets were imputed using MaCH software [32] up to ∼2.5 million SNPs based on the HapMap Phase I+II CEU release 22 (hg18/build36) reference.

### Gene Set Analysis

We analyzed SNPs within a 10-kb window around the genes encoding proteins belonging to the 5 DNA repair pathways described in the Kyoto Encyclopedia of Genes and Genomes (KEGG) pathway database [33], [34]; the BER pathway, the nucleotide excision repair (NER) pathway, the mismatch repair (MMR) pathway, the homologous recombination pathway and the NHEJ pathway. Gene set analyses were performed with the PLINK set-based test v1.07 in a case-control setting. [35] In the first step of this test, a single SNP analysis of all SNPs within the set is performed. Subsequently, a mean SNP statistic is calculated from the single SNP statistics of a maximum amount of SNPs below a certain P-value threshold. For the current study this threshold was set on 0.20 to ensure that all SNPs with minor effect will be analyzed. If SNPs are not independent, i.e. the LD (expressed in R^2^) is above a certain threshold, the SNP with the lowest P-value in the single SNP analysis is selected. This analysis is repeated with 10,000 simulated SNP sets, in which the phenotype status of the individuals is permuted. An empirical P-value for the SNP set is computed by calculating the number of times the test statistic of the simulated SNP sets exceeds that of the original SNP set. For the set-based analysis of this study, the parameters were set to P-value threshold <0.20, R^2^ threshold <0.5, and maximum number of SNPs = 99999. The associations were considered significant, after correction for the 5 analyzed pathways, if the P-value <0.01 (0.05/5). For the pathway analysis we only used the genotyped GWAS sets of both studies, since imputed SNPs were obtained based on their LD pattern with the genotyped SNPs and the LD threshold of the set-based analysis corrects for this, making their added value minimal.

## Results

Participant characteristics of the two study populations are presented in table 1. The main differences in the baseline characteristics between the two study populations are the mean age of the participants (GENDER; 62.5 years, PROSPER; 75.3 years) and the proportion of women (GENDER; 27%, PROSPER; 52%) Moreover, diabetes and complaints of stable angina pectoris were more frequent in GENDER, whereas a history of hypertension was more frequent in PROSPER. The incidence of MI in GENDER was 45% and in PROSPER 22%.

**Table 1:** Baseline characteristics and endpoints of the GENDER and PROSPER studies.

	GENDERN = 866	PROSPERN = 5,244
**Baseline characteristics**		
Age (years)	62.5±10.8	75.3±3.4
Male gender (N)	634 (73)	2,524 (48)
Current smoking (N)	216 (25)	1,392 (27)
History of diabetes (N)	177 (20)	544 (10)
History of hypertension (N)	349 (40)	3,257 (62)
History of angina (N)	288 (68)	1,424 (27)
History of myocardial infarction (N)	365 (42)	708 (14)
Total cholesterol (mmol/L)	4.9±1.0^a^	5.7±0.9
Body mass index (kg/m^2^)	27.0±3.7	26.8±4.2
Statin treatment	465 (54)	2,605 (50)
**Endpoints**		
Myocardial infarction^b^	389 (45)	1145 (22)
Stroke^b^	49 (6)	731 (14)
Myocardial infarction or stroke^b^	416 (48)	1714 (33)
Clinical restenosis^c^	295 (34)	NA
All cause mortality	237 (27)^d^	548 (11)^c^
Vascular mortality^c^	NA	266 (5)

Data are presented as mean ± SD or number (%).

a Cholesterol levels available in only 177 patients.

b Before inclusion or during follow-up period.

c During follow-up period.

d During follow up of 10 years after inclusion in GENDER.

Of the 5 selected DNA repair pathways, as described by the KEGG pathway database, the NER pathway was the largest one (44 genes), (Table S1), while the NHEJ pathway was the smallest (13 genes). The BER pathway consisted of 35 genes, the MMR pathway of 23 genes and the homologous recombination pathway of 28 genes.

The set-based analysis of the 5 pathway sets in the GENDER population resulted in a significant association of the homologous recombination pathway with MI (P = 0.011) and the combined endpoint of MI or stroke (P = 0.0039) (Table 2). A significant association with the same endpoints was also found for the NHEJ pathway (P = 0.0083 for MI and P = 0.0089 for MI or stroke). No significant association of any of the DNA repair pathways was found with stroke alone.

**Table 2:** Set-based analysis of DNA repair pathways in the GENDER and PROSPER study populations.

				MI^a^		Stroke^a^		MI or Stroke^a^	
	Pathway	Genes	SNPs	SNPs	P	SNPs	P	SNPs	P
GENDER^b^				N = 389/477		N = 49/817		N = 416/450	
Base excision		35	315	37	0.48	36	0.47	37	0.59
Nucleotide excision		44	401	57	0.38	49	0.34	60	0.38
Mismatch repair		23	285	39	0.34	40	0.42	47	0.35
Homologous recombination		28	418	64	**0.011**	51	0.71	60	**0.0039**
Non-homologous end joining		13	137	19	**0.0084**	14	0.43	19	**0.0089**
PROSPER^b^				N = 1,145/4,099		N = 731/4,513		N = 1,714/3,530	
Base excision		35	298	43	**0.049**	42	0.39	39	0.43
Nucleotide excision		44	392	62	0.34	57	0.47	49	0.49
Mismatch repair		23	285	39	0.43	33	0.46	31	0.91
Homologous recombination		28	426	61	0.14	42	0.49	51	0.24
Non-homologous end joining		13	144	19	**0.014**	30	0.35	22	0.11

The SNPs per endpoint indicate the number of independent SNPs that passed the test constrains (P<0.2 and R^2^<0.5) and were thus jointly analyzed in 10,000 permutations.

a Before inclusion or during follow-up period. MI, myocardial infarction.

b Cases/controls.

Analysis in the PROSPER dataset resulted in an association of the NHEJ pathway with MI (P = 0.014). A borderline significant association of the BER pathway with MI was also observed (P = 0.049). The other pathways did not show significant associations in PROSPER (Table 2).

To determine by which genes the association of the NHEJ pathway with MI was driven, we examined the SNP set from each gene of this pathway separately. We found that the association was driven by several genes from this pathway. In GENDER the *XRCC4* gene demonstrated a borderline significant association (P = 0.055) and in PROSPER the genes *PRKDC* (P = 0.029) and *LIG4* (P = 0.030) were individually associated with MI. The strongest association was found with the *MRE11A* gene in both GENDER (P = 0.0001) and PROSPER (P = 0.0017), driven by 3 and 2 SNPs, respectively, which differ between the studies (Table 3).

**Table 3:** Results of the gene set analysis of the non-homologous end joining pathway with myocardial infarction in GENDER and PROSPER.

	GENDER							PROSPER						
Gene	SNPs	Sig. SNPs	P (gene)	Top SNP	MAF	OR	P (SNP)	SNPs	Sig. SNPs	P (gene)	Top SNP	MAF	OR	P (SNP)
*XRCC5*	19	3	0.23	rs3821107	0.24	0.79	0.04	22	1	0.89	rs828704	0.20	0.93	0.19
*NHEJ1*	15	1	0.55	rs7588654	0.03	0.65	0.14	15	0	1.00				
*XRCC4*	29	4	**0.055**	**rs13178127**	**0.05**	**2.02**	**0.001**	30	4	0.38	rs35271	0.14	1.15	0.039
*RAD50*	8	0	1.00	–				10	1	0.52	rs2237060	0.44	1.07	0.13
*POLM*	4	2	0.12	rs11769882	0.25	0.79	0.040	4	1	0.26	rs11769882	0.22	0.93	0.14
*PRKDC*	14	1	0.69	rs7003908	0.34	1.16	0.14	14	4	**0.026**	**rs10109984**	**0.38**	**1.15**	**0.005**
*POLL*	3	0	1.00	–				3	1	0.28	rs3730477	0.21	1.09	0.14
*DCLRE1C*	8	1	0.22	rs12572872	0.23	1.24	0.06	8	0	1.00				
*DNTT*	9	1	0.35	rs1923703	0.12	0.80	0.14	9	0	1.00				
*MRE11A*	14	3	**0.0001**	**rs535801**	**0.31**	**1.52**	**0.00006**	13	2	**0.0017**	**rs2155209**	**0.35**	**0.86**	**0.002**
*FEN1*	5	0	1.00	–				5	1	0.27	rs695867	0.35	0.80	0.10
*LIG4*	7	3	0.31	rs9520823	0.30	1.21	0.07	7	3	**0.030**	**rs1151403**	**0.42**	**0.87**	**0.003**
*XRCC6*	2	0	1.00	–				4	1	0.45	rs17002523	0.01	1.34	0.16

SNPs, total number of SNPs per gene; Sig.SNPs indicate the number of SNPs that passed the test constrains (P<0.2 and R2<0.5) and were thus jointly analyzed in 10,000 permutations; OR odds ratio; P, p-value; MAF, minor allele frequency.

**Figure 1:**
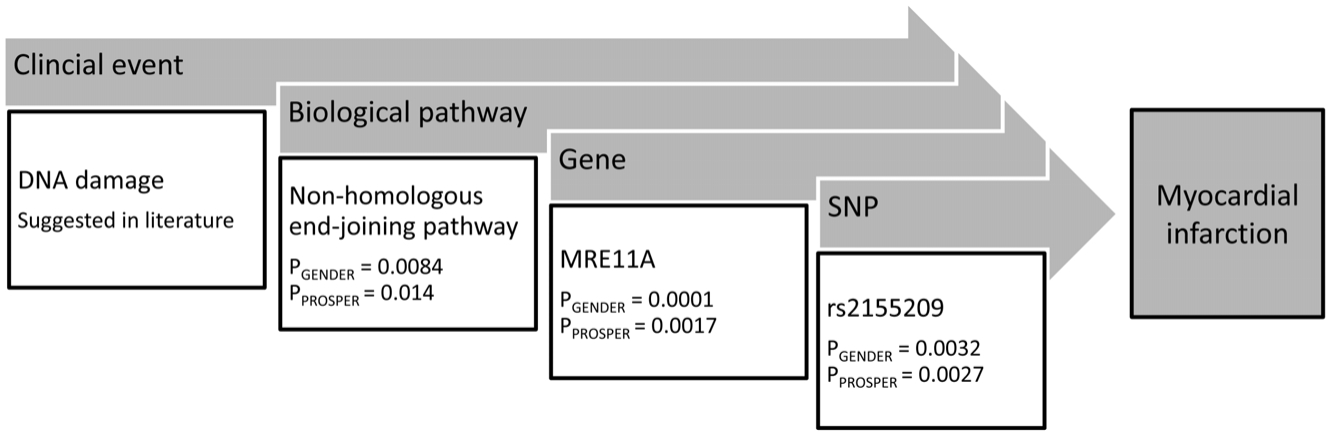
Fine mapping from DNA damage through the identification of an associated DNA repair pathway, the responsible gene in this pathway, to the single nucleotide polymorphism (SNP).

By using imputed data we performed in silico fine mapping of the individual SNPs in the *MRE11A* genetic region on chromosome 11 (Figure 1). Within the range of 10 Kb around the *MRE11A* gene genotypic data of 104 SNPs were available. We identified 8 sets of SNPs that were in high LD (R^2^>0.8) but only one set (10 SNPs) associated with MI in both GENDER and PROSPER (P = 0.0033 and P = 0.0023 respectively). This set contained the top SNP in *MRE11A* in PROSPER (rs2155209), that was identified in the individual gene analysis (Table 4). This SNP is located in a DNase I hypersensitivity site (UCSC genome browsers database [36]). The promotor 2.0 Prediction Server [37] reported that the region surrounding rs2155209 is not a promotor region. In addition, the is-rSNP algorithm [38] reported that the DNA binding affinity of three transcription factors is significantly affected by rs2155209 (LM221 P = 0.014, estrogen receptor 2 (ESR2) P = 0.028 and LM168 P = 0.037). Unfortunately, rs2155209 is not reported in three publically available eQTL databases (mRNA by SNP browser [39], [40], VarySysDB [41] and the eQTL database of the Pritchard lab [42]).

**Table 4:** Genomic region of MRE11A divided in LD blocks for the association with myocardial infarction.

					GENDER		PROSPER	
Set	SNPs	R^2^	Tagging SNP	MAF*	OR (95% CI)	P	OR (95% CI)	P
1	10	0.91	**rs2155209**	**0,36**	**0.74 (0.61–0.91)**	**0,0033**	**0.86 (0.78–0.96**	**0,0023**
2	10	0,90	rs535801	0,31	1.52 (1.24–1.87)	0,000059	1.00 (0.90–1.11)	0.94
3	17	0.88	rs1270146	0.43	1.32 (1.09–1.60)	0.0049	1.02 (0.93–1.13)	0.63
4	13	0.91	rs529126	0.26	1.44 (1.16–1.80)	0.00090	1.02 (0.91–1.12)	0.71
5	5	0.85	rs499952	0.35	1.32 (1.08–1.61)	0.0059	1.03 (0.92–1.12)	0.54
6	4	0.91	rs13447720	0.23	0.94 (0.75–1.18)	0.57	1.18 (1.05–1.30)	0.0027
7	18	0.91	rs10765682	0.09	0.91 (0.65–1.27)	0.58	1.01 (0.87–1.20)	0.95
8	18	1.00	rs12788248	0.01	1.22 (0.43–3.49)	0.71	1.02 (0.58–1.72)	0.95
other**	9		–					

R^2^ indicates lowest LD between SNPs within the set.

Tagging SNP is a genotyped SNP with the lowest P-value per set.

* MAF, minor allele frequency in GENDER.

** SNPs not in LD with other SNPs within the gene.

To explore whether the found associations were caused by specific subgroups we analyzed the NHEJ pathway in male and female patients, smokers and non-smokers and in patients with and without diabetes separately. Moreover, in the PROSPER study we also performed the analyses in the pravastatin and placebo group. No clear associations were detected in these subgroups (Table S2).

## Discussion

The current study is the first to use a DNA repair pathway approach for the identification of new candidate genes related to cardiovascular outcomes. We show that genetic variation in several of these genes are indeed associated with cardiovascular related endpoints and that the joint analyses of these genetic markers demonstrates a significant association of the NHEJ pathway with prevalent and incident MI in two study populations. Variation in the gene encoding meiotic recombination 11 homolog A (*MRE11A*) drives the association.

This is the first study that demonstrates a relation between the NHEJ pathway and MI or even with CVD. This particular DNA repair pathway is mainly involved in the repair of double-strand breaks (DSBs), which are considered to have the highest risk of evoking deleterious events, such as chromosomal translocations, cancer and cell death. The main driver of this association, MRE11A, is a highly conserved protein, existing in vivo as a dimer, forms together with RAD50 and NBS1 the MRN complex. [43] The MRN complex has a critical role in the recognition of DNA damage lesions or the chromatin alterations that follow DNA damage [3] and a key role in the cellular response to DSBs. [44] Moreover, the MRN complex has been implicated in telomere maintenance, meiosis, DNA replication and checkpoint activation. [45]–[47] Genetic variation in *MRE11A* has previously been associated with several types of cancer [48]–[50] and antaxia-telangiectasis-like disease. [51] To our knowledge, no association of this gene with CVD events has yet been described, also not in the previous GWAS on CVD. [23], [52], [53] The SNP rs2155209, significantly associated with MI in both of our study populations, has been associated with an 1.5-fold increased risk of bladder cancer, although the authors of that study suggest that it might has been a false-positive finding. [49] This *MRE11A* SNP is located in the 3′UTR of the gene, and the possible functional effect has not yet been studied.

When examining the LD structure of *MRE11A*, we found that the structure is not conform the expected LD block formation, meaning that nearby SNPs are organized into regions of high LD separated by short segments of very low LD. This discontinuous LD structure is not uncommon, and has been described before for this gene. [54] Allen-Brady et al. [54] describe 4 tagging SNPs together accounting for 99% of the genetic variance within the gene region. In the current study the genetic coverage of the gene was more thorough (104 SNPs compared to 11 in the former study), resulting in 8 tagging SNPs. Interestingly, one of the 4 described tagging SNPs, rs556477, is in very high LD (R^2^ = 0.92) with our top SNP rs2155209. It is unknown whether rs2155209 has any direct functional effects. That this SNP is located in a DNase I hypersensitivity site, which often associated with *cis*-regulatory sequences, including promoters, insulators, enhancers and locus control regions, increases the likelihood that rs2155209 influences one of these features and thereby exerting its clinical effects, although this remains to be proven. Another method of predicting the possible regulatory abilities of non-coding SNPs is the in silico rSNP algorithm [38]. This approach indicated that rs2155209 affects binding of ERS2. The estrogen receptor 2 belongs to a family of nuclear receptor transcription factors, activating transcription upon binding to specific DNA sequences. Moreover, the SNP was associated with LM221 and LM168, two conserved motifs in the human genome described to be involved in gene regulation, likely serving as insulators. [55] Wet lab confirmation of these bioinformatic predictions will be necessary before definite conclusions can be drawn from these findings.

Cigarette smoking is considered to be an important risk factor in atherosclerotic vascular disease and it is a well-known external factor associated with DNA damage, like single- and double strand breaks and the formation of oxidative DNA-adducts. [56], [57] The association of the NHEJ pathway with MI, demonstrated in the current study, could indicate that this pathway is the underlying mechanism of the strong relation between smoking and CVDs. However, this hypothesis could not be confirmed in subgroup analyses. Whether this absence of association is caused by lack of power of the subgroup analysis, or because the underlying mechanism causing MI is not through smoking, is uncertain. Moreover, the hypothesis that patients with diabetes mellitus have increased oxidative stress which could lead to DNA damage [58], [59], could also not be confirmed in our population. However, considering that the incidence of diabetes was 20% in GENDER and only 10% in PROSPER, there was not enough power to detect a small effect. Furthermore, we cannot exclude that another DNA repair pathway than NHEJ might be responsible for the DNA repair in diabetic patients, but considering the small subgroup size and the fact that the other DNA repair pathways were not significantly associated in the complete populations, we did not perform further analyses for these pathways. The subgroup analysis did demonstrate that the association of the NHEJ pathway with MI was possibly driven by the male subjects, since in PROSPER no association was found in female subjects. Although in GENDER a similar trend was observed, these results were not significant.

The strength of gene set analysis, opposed to GWAS analysis, is that it tests the joint effect of multiple individual SNPs within a larger set. Considering the a priori small effect size of the individual SNPs on complex disease endpoints, like MI, analysis of the joint effect of multiple markers, in this study comprising complete DNA repair pathways, will increase the likelihood of finding biological plausible associations.

Several possible limitations to our study have to be mentioned. For the current study we performed the pathway analysis using the PLINK software. [60] Other software packages have been described, although to date none has been proven to be clearly superior to the others. Gui and colleagues compared 7 tests analyzing the WTCCC Crohn’s Disease dataset. [61] One of their overall conclusions was that the set-based test in PLINK was the most powerful algorithm. Another study, applying PLINK set-based test, Global test, GRASS and SNP ratio test, for the analysis of three pathways regarding human longevity observed similar results with the different tests. [62] Although other software packages could lead to different results, the fact that our fine mapping strategy led to the identification of a single LD block associated in two independent populations, increased the likelihood of a true positive association.

The 5 DNA repair pathways analyzed were derived from the publically available KEGG database [34]. The KEGG database is however not the only database providing biological pathways and there is no consensus on the best database. In our opinion these particular pathways were more elaborately described by KEGG than in other databases (for instance Reactome or BioCarta). Moreover, only the KEGG database provided a description of all 5 DNA repair pathways. Since the overlap of certain pathways of different databases is substantial, we decided only to test the DNA repair pathways described by KEGG. [33] It is important to realize that probably none of these databases provide a perfect representation of the actual biological mechanism, simple because our current knowledge is not that far evolved yet. Likely not all genes incorporated within the current pathways directly influence the actual DNA repair process of interest. These unrelated gene product could therefore interfere with the actual associations, however to what extent this is the case in the current study remains unknown.

As stated above, DNA damage and DNA damage repair are associated with cancer. Since we are interested in the effects of DNA damage repair on clinical events other than cancer, and because the two included study populations are of rather old age, especially PROSPER, we cannot exclude that the competing risk of cancer related mortality and CVD events have led to a selection bias of the patients. Therefore, it could be possible that the role of DNA repair pathways is being underestimated. However, since we cannot correct for this potential selection bias, this remains speculative. Another potential confounder is age. The GENDER study is considerably younger than the PROSPER cohort, possibly explaining part of the different results of both studies. However, since in the set-based analysis of PLINK correction for confounders is not possible, the actual magnitude of the influence of age on the current results remains therefore uncertain.

In conclusion, with this study we demonstrate that genetic variation in the NHEJ pathway of the human DNA repair machinery, and specifically genetic variation in the *MRE11A* gene, is associated with the occurrence of MI. Results of this study need to be validated by functional studies to further elucidate the precise mechanistic role of NHEJ in atherosclerotic lesion formation.

## Supporting Information

Table S1 Characteristics of the 5 DNA repair pathways.
(XLS)

Table S2 Subgroup analysis of the non-homologous end-joining pathway in GENDER and PROSPER.
(XLS)
